# Intestinal Microbiota Participates in the Protective Effect of HO-1/BMMSCs on Liver Transplantation With Steatotic Liver Grafts in Rats

**DOI:** 10.3389/fmicb.2022.905567

**Published:** 2022-06-10

**Authors:** Mengshu Yuan, Ling Lin, Huan Cao, Weiping Zheng, Longlong Wu, Huaiwen Zuo, Xiaorong Tian, Hongli Song

**Affiliations:** ^1^Tianjin First Central Hospital Clinic Institute, Tianjin Medical University, Tianjin, China; ^2^Department of Organ Transplantation, Tianjin First Central Hospital, Tianjin, China; ^3^National Health Commission (NHC) Key Laboratory of Critical Care Medicine, Tianjin, China; ^4^School of Medicine, Nankai University, Tianjin, China; ^5^Tianjin Key Laboratory of Organ Transplantation, Tianjin, China

**Keywords:** bone marrow mesenchymal stem cells, liver transplantation, steatotic liver graft, tight junction (TJ), intestinal permeability, intestinal microbiota

## Abstract

The present study aimed to explore whether heme oxygenase-1 (HO-1)-modified bone marrow mesenchymal stem cells (BMMSCs) have a protective effect on liver transplantation with steatotic liver grafts in rats, and to determine the role of the intestinal microbiota in such protection. HO-1/BMMSCs were obtained by transduction of *Hmox1* gene [encoding heme oxygenase (HO-1)]-encoding adenoviruses into primary rat BMMSCs. Steatotic livers were obtained by feeding rats a high-fat diet, and a model of liver transplantation with steatotic liver grafts was established. The recipients were treated with BMMSCs, HO-1/BMMSCs, or neither, *via* the portal vein. Two time points were used: postoperative day 1 (POD 1) and POD 7. The results showed that under the effect of HO-1/BMMSCs, the degree of steatosis in the liver grafts was significantly reduced, and the level of liver enzymes and the levels of pro-inflammatory cytokines in plasma were reduced. The effect of HO-1/BMMSCs was better than that of pure BMMSCs in the prolongation of the rats' postoperative time. In addition, HO-1/BMMSCs promoted the recovery of recipients' intestinal structure and function, especially on POD 7. The intestinal villi returned to normal, the expression of tight junction proteins was restored, and intestinal permeability was reduced on POD 7. The intestinal bacterial of the LT group showed significantly weakened energy metabolism and overgrowth. On POD 1, the abundance of *Akkermansiaceae* was higher. On POD 7, the abundance of *Clostridiaceae* increased, the level of lipopolysaccharide increased, the intestinal mucosal barrier function was destroyed, and the levels of several invasive bacteria increased. When treated with HO-1/BMMSCs, the energy metabolism of intestinal bacteria was enhanced, and on POD 1, levels bacteria that protect the intestinal mucosa, such as *Desulfovibrionaceae*, increased significantly. On POD 7, the changed intestinal microbiota improved lipid metabolism and increased the levels of butyrate-producing bacteria, such as *Lachnospiraceae*. In conclusion, HO-1/BMMSCs have protective effects on steatotic liver grafts and the intestinal barrier function of the recipients. By improving lipid metabolism and increasing the abundance of butyrate-producing bacteria, the changed intestinal microbiota has a protective effect and prolongs the recipients' survival time.

## Introduction

Liver transplantation is the most effective treatment for end-stage liver disease and liver failure. The shortage of donor livers and the deterioration of the quality of donor livers restricts the widespread application of liver transplantation. With changes in lifestyle and the normalization of high-fat and high-sugar diets, the global incidence of fatty liver has reached as high as 25% (Sheka et al., 2020). As a result, the use of steatotic donor livers (especially moderately steatotic livers) is increasingly common in liver transplantation, accounting for 30 and 20% of cadaveric and living donor livers, respectively (Jackson et al., 2020). However, grafts using severely steatotic livers will directly affect the postoperative survival time of liver transplant recipients (Hughes and Humar, 2021). Therefore, to improve the quality of life and prolong the survival time of liver transplantation recipients, it is very important to develop a method of repairing steatotic liver grafts.

Mesenchymal stem cells (MSCs) have the potential for self-replication and multi-directional differentiation, and can be inserted into injured tissues to replace damaged cells. Their paracrine effects have been used in tissue repair, regeneration, and immune regulation of the body (Yang et al., 2014; Mahrouf-Yorgov et al., 2017; Sun et al., 2020b). Bone marrow mesenchymal stem cells (BMMSCs) have been transfected with genes such as *HMOX1* [encoding heme oxygenase-1 (HO-1)] to form HO-1/BMMSCs, which could increase the activity and efficiency of BMMSCs. Increasing the local survival time of BMMSCs, and enhancing their anti-apoptotic, anti-inflammatory, and anti-oxidative stress functions, can also improve their repair and immune regulation capabilities (Zheng et al., 2017; Chen et al., 2019; Sun et al., 2020a). However, studies on stem cells and steatotic liver transplantation are few and lack detail.

The liver-gut axis plays an important role in the occurrence and development of non-alcoholic fatty liver disease, and portal blood flow and bile acids exert a bridge function to connect the liver and the intestines (Adolph et al., 2018; Albillos et al., 2020). Dysbiosis and an impaired intestinal mucosal barrier lead to bacterial translocation and the entry of their metabolites into the bloodstream, causing chronic low-grade inflammation throughout the body, and are closely related to the occurrence and development of non-alcoholic fatty liver disease (Lang and Schnabl, 2020; Tilg et al., 2020; Yang et al., 2020). Microbially-induced differences might impact the course of cholestasis and modulate liver injury (Juanola et al., 2021). Emerging data demonstrate that gut-derived lipopolysaccharide, gut microbiota-associated bile acids, and other bacterial metabolites, such as short-chain fatty acids and tryptophan metabolites, might play multifaceted roles in liver injury and regeneration (Zheng and Wang, 2021). In addition, the gut microbial signature of impaired inhibitory control, which is associated with addictive disorders that can lead to cirrhosis, is distinct from cirrhosis-related cognitive impairment (Bajaj et al., 2021). At the same time, the intestinal microbiota is also involved in the repair of recipients after liver transplantation (Mu et al., 2019; Nakamura et al., 2019; Sharpton et al., 2021). Therefore, the present study sought to determine whether HO-1/BMMSCs could protect steatotic liver grafts and play a role in alterations of the intestinal microbiota, aiming to explore the relationship between liver transplantation with steatotic liver grafts and the intestinal microbiota under the action of HO-1/BMMSCs. We believe that the results will provide a favorable foundation and research direction to improve the prognosis of steatotic liver transplantation.

## Materials and Methods

### Animals and Steatotic Liver Grafts Model

The experiments were performed using specific pathogen-free (SPF) rats (China Food and Drug Administration, Beijing, China). Male SD rats, 6–7 weeks old, weighing 180–200 g, were fed a high-fat diet (15% fat, 15% sucrose, 10% egg yolk powder, 1% cholesterol, 0.2% bile salts, 58.8% basal diet, Chinese food Drug Administration, Beijing, China). After 12 weeks, they had reached a weight of 450–500 g, and liver oil red and hematoxylin and eosin (H&E) staining were used to estimate whether the rat livers were steatotic. The steatotic livers were used as grafts. Male SD rats were fed with a normal basal diet for 22–24 weeks until they weighed 450–500 g, at which point, they were used as the steatotic liver transplant recipients. All animal experiments followed the Guidelines for the Care and Use of Laboratory Animals and were approved by the Animal Ethics Committee of Nankai University (Permit number: 2021-SYDWLL-000331).

### BMMSCs Culture, Preparation, and Identification of HO-1/BMMSCs

Isolation, culture, identification, and gene transfection of BMMSCs were performed as previously described (Yin et al., 2017). Bone marrow contents and BMMSCs were obtained from rat femurs and tibias by repeated pipetting. HO-1/BMMSCs were obtained by transfecting BMMSCs with adenoviral vectors (Ad/HO-1; Shanghai Jikai Gene, Shanghai, China) expressing Green fluorescent protein (GFP) and/or *Hmox1* constructs. The molecular biological functions of the cells were identified by inducing adipogenic and osteogenic differentiation *in vitro*. Cell phenotyping was performed using flow cytometry to identify cluster of differentiation (CD)29, CD90, RT1A (rat MHC class I antibody), CD34, CD45 and RT1B (rat MHC class II antibody) (BioLegend, San Diego, CA, USA) expression. Immunofluorescence, western blotting, and quantitative real-time reverse transcription PCR (qRT-PCR) were used to detect the expression of HO-1 in cells.

### Establishment of the Rat Liver Transplantation Model

Liver transplantation was performed as described previously (Cao et al., 2020). Rats were anesthetized, the liver was exposed, and heparin (1 U/g) was injected. After 10 min of heparinization, the diaphragm was incised, the thoracic artery was clamped using an arterial clip, the heart was compressed, and cardiac arrest was induced. The abdominal cavity was then covered with warm saline at 37°C for 30 min. During this period, a temperature-sensing probe was placed in the rats' abdominal cavity to detect and maintain the body temperature between 35 and 37°C. According to the suggestion by Kamada and Calne (Kamada and Calne, 1979), the same surgeon performed all the operations. The duration of the anhepatic phase was 30 ± 1 min.

According to the different treatment methods of the steatotic donor livers, recipients were divided into four groups, namely the sham operation group (Sham group), the liver transplantation with steatotic liver grafts group (LT group), the LT + BMMSCs treated group (BM group), and the LT + HO-1/BMMSCs treated group (HBM group). The steatotic liver grafts in the BM group were perfused with about 1 × 10^7^ BMMSCs *via* the portal vein, and those in the HBM group were perfused with about 1 × 10^7^ HO-1/BMMSCs. Five animals in the Sham group were used for blood, liver, and intestinal histopathological specimens, and ileocecal feces (about 0.2 g) collection. Ten animals in each experimental group were used for blood, liver, and intestinal histopathological specimens, and ileocecal feces (about 0.2 g) collection on post operational day (POD) 1 (*n* = 5 in each group) and POD 7 (*n* = 5 in each group), respectively. Twenty-four (*n* = 6 in each group) animals were used for survival analysis; 40 animals (*n* = 10 in each group) were used for biochemical analysis; and three animals were used for BMMSC *in vivo* tracking.

### Establishment of an Injury Model of Steatotic IAR20 Cells

After stimulating steatosis of IAR20 cells (National Infrastructure of Cell Line Resource, Beijing, China) with 100 μM palmitic acid (Sigma-Aldrich, St. Louis, MO, USA) and 200 μM oleic acid (Sigma), lipopolysaccharide (LPS) at 10 μg/mL used to treat the steatotic IAR20 cells for 24 h to establish the steatosis hepatocyte injury model. According to the different treatment methods of the IAR20 cells, they were divided into four groups: ➀ The control group; ➁ the LPS group (steatotic IAR20 cells stimulated with 10 μg/mL LPS for 24 h); ➂ the LBM group (after obtaining the LPS-injured steatotic IAR20 cell model, 1 × 10^6^ BMMSCs/well were added, and co-cultured for 24 h); and ➃ the LHM group (after obtaining the LPS-injured steatosis IAR20 cell model, 1 × 10^6^ HO-1/BMMSCs/well were added, and co-cultured for 24 h). The activity of IAR20 cells and the mRNA and protein levels of Toll-like receptor 4 (TLR4) were determined.

### Establishment of an Injury Model of Intestinal Epithelial Cells

The 10 μg/mL LPS was used to treat IEC-6 and Caco-2 cells (National Infrastructure of Cell Line Resource, Beijing, China) for 24 h to establish intestinal epithelial cell injury models. After obtaining the LPS-injured intestinal epithelial cell model, 1 × 10^6^ BMMSCs were added to the upper layer of a 0.8-μm Transwell chamber (Corning Inc., Corning, NY, USA), and the lower layer comprised intestinal epithelial cells. The cells in the chambers were co-cultured for 24 h, to rule out the role of MSCs in direct contact with intestinal epithelial cells. The activity of intestinal epithelial cells and the levels of tight junction proteins were determined. According to the different treatment methods of intestinal epithelial cells, they were divided into the same for groups as the injury model of steatotic IAR20 cells.

### Liver Biochemical Examination

Plasma was taken and an automatic biochemical analyzer (Cobas 800, Roche, Basel, Switzerland) was used to detect serum alanine aminotransferase (ALT), aspartate aminotransferase (AST), alkaline phosphatase (ALP), glutamyl transpeptidase (GGT), total bilirubin (TBil), and serum albumin (ALB). The assays were carried out according to the instrument manual.

### Histopathology

The liver tissue of the left lateral lobe, with a size of 2 × 2 × 1 cm^3^, and ileum tissue at about 6–8 cm away from the ileocecal area were sampled, completely immersed in 10% neutral formalin solution, fixed for 48 h, and then embedded in parafilm. Serial 3 μm sections were obtained and stained using hematoxylin and eosin (H&E).

### Transmission Electron Microscopy (TEM)

Fresh ileocecal intestinal tissue was cut into 1 × 1 × 2 mm^3^, preserved with 2.5% glutaraldehyde, fixed with epoxy resin, and cut into ultrathin sections. The sections were observed using a transmission electron microscope (HT7800, Hitachi, Tokyo, Japan).

### Enzyme-Linked Immunosorbent Assay (ELISA)

Whole blood samples were kept at 4°C for 30 min, centrifuged at 4°C (500 × *g* for 20 min), and the supernatant was taken and stored in a −80°C refrigerator. Measurements of serum interleukin-1β (IL-1β), IL-6, IL-10, tumor necrosis factor-α (TNF-α), diamine oxidase (DAO), D-lactic acid (D-LA), and LPS levels were carried out using ELISA kits (Multisciences Biotech Co., Hangzhou, China) according to the manufacturer's instructions.

### Immunofluorescence

Rat ileum samples taken at about 2–3 cm away from the ileocecal region were collected, placed in optimal cutting temperature compound (OCT) for embedding, and immediately placed in a −80°C refrigerator for long-term storage. Frozen tissues were sectioned, fixed, and sealed, and then incubated with anti-zona occludens 1 (ZO-1) (1:250) and anti-Occludin (1:250) (Cell Signaling Technology, Danvers, MA, USA) primary antibodies at 4°C overnight in the dark. Next day, the sections were incubated secondary antibodies (1:250) at room temperature for 60 min in the dark, and then the nuclei were counterstained using 4′,6-diamidino-2-phenylindole (DAPI) solution by incubation in the dark for 5 min. The sections were stored at 4°C, and photographed under a fluorescence microscope.

### Western Blotting

Total protein was extracted by high performance liquid chromatography into radioimmunoprecipitation assay (RIPA) buffer with added phenylmethanesulfonyl fluoride (PMSF). The total protein concentration was determined using a bicinchoninic acid (BCA) assay. The proteins were separated by electrophoresis using sodium dodecyl sulfate-polyacrylamide gel electrophoresis (SDS-PAGE), transferred to polyvinylidene fluoride (PVDF) membranes, and blocked using 5% skim milk for 1 h. The membranes were then incubated with primary antibody overnight at 4°C. The primary antibodies used included those recognizing: TLR4 (1:1000) and β-actin (1:3000) (Multisciences Biotech Co., Hangzhou, China). After overnight incubation, the membranes were rinsed with TBST buffer (Tris buffer, NaCl, and Tween 20) and incubated with the corresponding secondary antibodies (1:2000) for 1 h at room temperature. Membranes were scanned using the ChemiDoc XRS+ imaging system (Bio-Rad, Hercules, CA, USA), and the gray values of the immunoreactive protein bands were analyzed using ImageJ 7.0 software (NIH, Bethesda, MD, USA). Relative protein expression levels were calculated in comparison with the level of β-actin.

### Quantitative Real-Time Reverse Transcription Polymerase Chain Reaction (qRT-PCR)

Total RNA from liver and intestinal tissues was extracted using the TRIzol reagent (Takara Biotechnology, Shiga, Japan), and cDNA was produced from the total RNA using a cDNA reverse transcription kit (Takara Biotechnology) according to the manufacturer's instructions. The cDNA was used as the template in the qPCR step of the qRT-PCR protocol. β-actin was used as an internal control. Data processing was performed using the 2^−ΔΔCT^ method for relative quantification (Livak and Schmittgen, 2001).

### Cell Counting Kit 8 (CCK8) Assay

**1**0 μg/mL LPS was used to treat IAR20, Caco-2, and IEC-6 cells for 24 hours in 96-well plates. The cells were then assayed using a CCK8 kit according to the manufacturer's instructions (Solarbio, Beijing, China). After the reaction was completed, the absorbance was measured at 450 nm using a microplate reader, and the cell viability was calculated:


$$\begin{array}{l} \text{Cell viability}\left(\text{\%}\right)\;=\;\left(\text{experimental group}-\text{blank}\right)/\\ \left(\text{control group}-\text{blank}\right) \end{array}$$

### 16S rRNA Sequencing

About 0.2 g of fresh fecal samples were collected from the rats in each group, placed in a closed sterile cryopreservation tube, and stored in liquid nitrogen. High-throughput sequencing of the 16S rDNA gene V3-V4 region was carried out by Beijing Boao Jingdian Co., Ltd. (Beijing, China) using the Illumina NovaSeq sequencing platform. Cutadapt (version 1.18) was used to trim and filter the original sequences to get the optimized number of clean tags. Clustering was performed according to 97% similarity sequences using USEARCH (Version 11.0.667) software to obtain the species distribution information of each sample. Based on the operational taxonomic units (OTU) analysis results, the species richness information of each sample was obtained.

The QIIME software was used to analyze the abundance information at different classification levels in the Silva database. The Rarefaction curve, species accumulation curves, and alpha diversity reflected by the Shannon index were analyzed using the Mothur software. Beta diversity analysis, reflected by principal component analysis (PCA) used the vegan and GUniFrac packages in R. In the LEfSe (Linear Discriminant Analysis (LDA) Effect Size) analysis, the nonparametric factor Kruskal–Wallis rank-sum test and the Wilcoxon rank sum test were used to analyze the differences between groups. We used the PICRUSt (Phylogenetic Investigation of Communities by Reconstruction of Unobserved States) software to predict the relative abundance of microbial functional categories in samples from 16S rDNA gene sequencing data. The R package limma was used to test the significant difference between the two groups for the prediction results of PICRUSt2.

### Data Analysis

SPSS 13.0 (SPSS GmbH, Munich, Germany) and GraphPad 8.0 (GraphPad Software, Inc., San Diego, CA, USA) were used for statistical analysis. Data are presented as the mean ± the standard deviation. One-way analysis of variance (ANOVA) was used to test the significance of the data. Count data were expressed as percentages (%) and the chi-squared test was used to assess data significance. Survival analysis was performed using Kaplan–Meier survival curves and log-rank tests (Mantel–Cox). Pearson's correlation method was used to determine the correlation coefficient (r) between groups. *P* < 0.05 was considered statistically significant.

## Results

### Phenotypic and Functional Identification of BMMSCs and HO-1/BMMSCs

BMMSCs are adherent growing cells, and there was no morphological change after transfection with Ad/HO-1, showing typical long fusiform or spindle shapes, and a swirling arrangement (Supplementary Figure 1A). Furthermore, HO-1/BMMSCs were osteogenic (Supplementary Figure 1B) and adipogenic (Supplementary Figure 1C). Flow cytometry results showed more than 98% CD29, CD90, and RT1A positivity, and more than 98% CD34, CD45, and RT1B negativity (Supplementary Figures 1D–F). Red fluorescence, indicating expression of HO-1 in BMMSCs (Supplementary Figure 1G) and HO-1/BMMSCs (Supplementary Figure 1H), was significantly higher in HO-1/BMMSCs than that in BMMSCs. The HO-1 protein level HO-1/BMMSCs was significantly higher than that in BMMSCs (*P* < 0.05), and the mRNA level of HO-1 gene (*Hmox1*) was also significantly higher than that in BMMSCs (*P* < 0.05) (Supplementary Figure 1I). Hence, HO-1 was successfully transfected into BMMSCs without affecting the molecular biological properties of BMMSCs.

### Morphology, Cell Distribution, and Survival Rate of Recipients Under the Function of HO-1/BMMSCs

**Figure 1:**
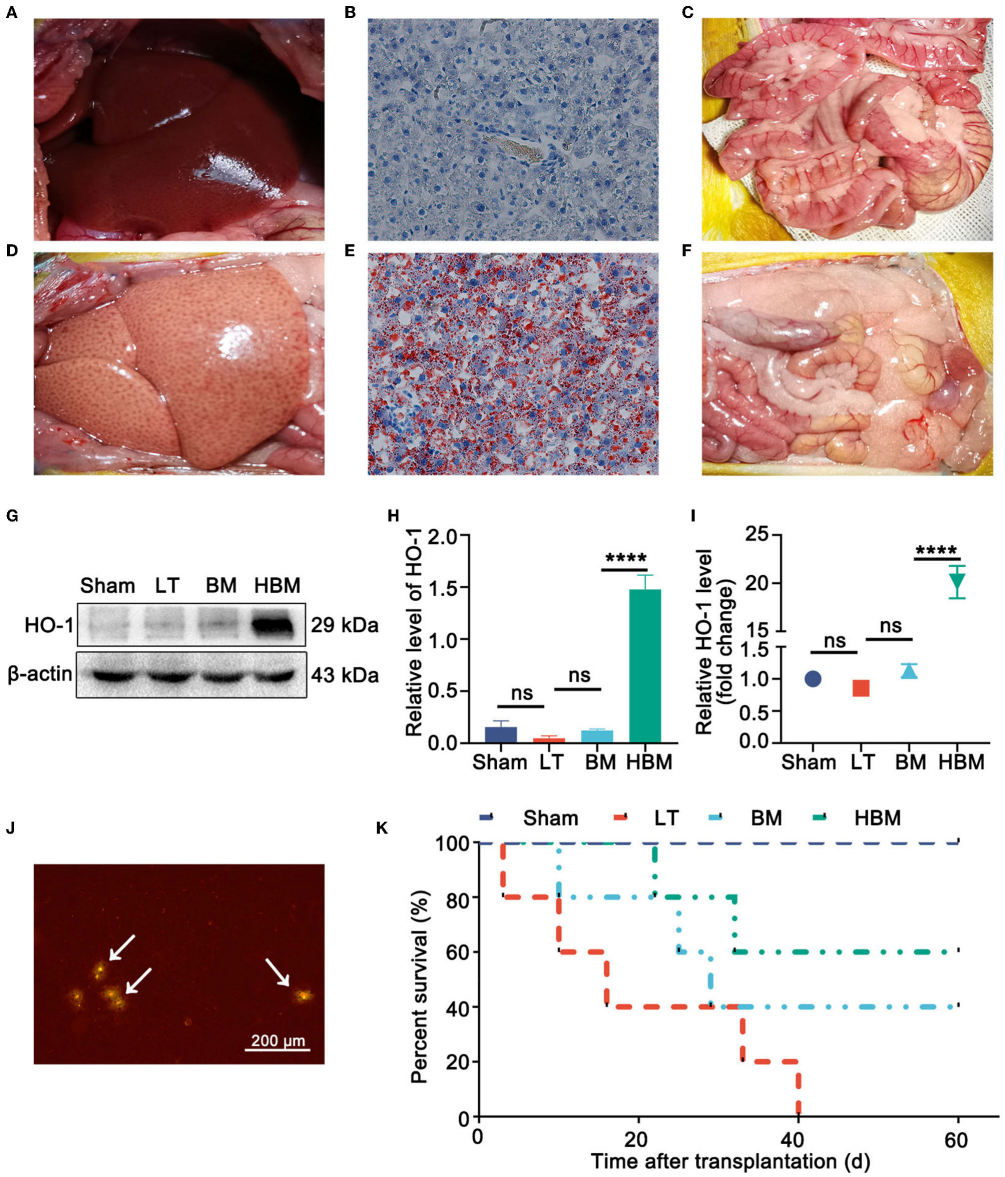
Steatotic liver graft morphology, stem cell distribution, and recipient survival. **(A)** Normal rat liver. **(B)** Oil red staining of a normal liver. **(C)** Normal rat gut. **(D)** Rat steatotic graft liver. **(E)** Oil red staining of steatotic liver grafts (×200). **(F)** Fat in the intestine of a donor rat. **(G,H)** On POD 7, the level of the HO-1 protein was the highest in the HBM group. **(I)** On POD 7, the expression of *Hmox1* mRNA was obvious in the HBM group. **(J)** Liver tissue frozen section of a steatotic donor liver (×40). GFP/BMMSCs colonization in the hepatic sinusoids could be observed by fluorescence microscopy. Arrows point to BMMSCs. **(K)** Survival time after liver transplantation for the steatotic donor livers. The median survival times of the Sham, LT, BM, and HBM groups were >60, 16, 29, and 60 d, respectively. *P < 0.05, **P < 0.01, ***P < 0.001, ****P < 0.001.

Morphological changes of transplanted livers in each group were as follows: normal rat livers appeared red with sharp edges (Figure 1A), had almost no oil red staining positive areas (Figure 1B), and only a small amount of fat in the mesentery (Figure 1C). By contrast, the steatotic liver graft's capsule was tense and smooth, generally yellow, with dull edges and a greasy appearance (Figure 1D); the positive area of oil red staining was significantly increased (Figure 1E), and obvious fat is seen in the mesentery (Figure 1F), indicating that steatotic liver grafts were successfully established.

The distribution of MSCs in the liver were as follows: on POD 7, the expression of HO-1 protein (Figures 1G,H) and mRNA (Figure 1I) in the liver tissue of the HBM group increased significantly (*P* < 0.05), which indirectly indicated that HO-1/BMMSCs were present in the liver and persisted. In the frozen sections of the steatotic liver grafts, an obvious red fluorescence signal for BMMSCs was observed, indicating that both BMMSCs and HO-1/BMMSCs could colonize the transplanted liver (Figure 1J).

From the survival curve, the median survival time of the HBM group was the longest (>60 d), while the median survival time of the LT group was the shortest (16 d). The median survival time of the BM group was 29 days, which was slightly higher than that of the LT group, but significantly lower than that of the HBM group (*P* < 0.05). Therefore, HO-1/BMMSCs could significantly prolong the survival time of steatotic liver transplantation recipients, and their effect was significantly better than that of BMMSCs alone (Figure 1K).

### HO-1/BMMSCs Can Improve the Function of the Steatotic Liver and Reduce Inflammation

**Figure 2:**
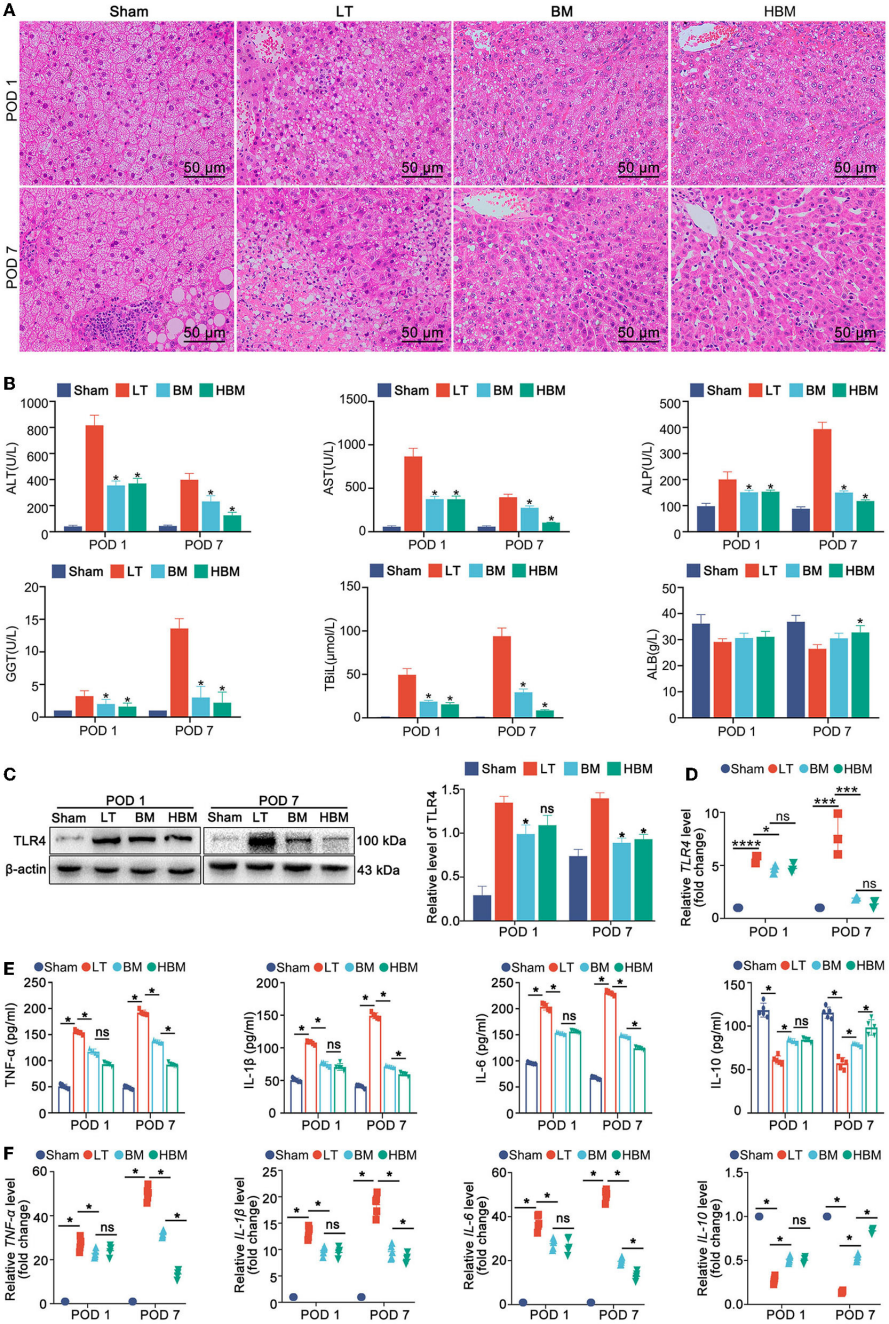
Changes in the steatotic liver graft histology, liver biochemical examination, and plasma inflammatory indexes in each group. **(A)** Histopathology of transplanted livers at different time points (H&E staining, ×200). **(B)** Transplanted liver function. On POD 1, the ALT level in the LT group (817.20 ± 77.63 U/L) was significantly higher than that in the BM group (355.70 ± 34.04 U/L) and the HBM group (370.60 ± 38.63 U/L) (*P* < 0.05), while the difference between the BM group and the HBM group was not statistically significant. On POD 7, the ALT level in the LT group (399.20 ± 47.56 U/L) was still significantly higher than that in the BM (232.60 ± 43.92 U/L) and HBM groups (126.00 ± 23.50 U/L), with a significant difference (*P* < 0.05). Changes in AST, ALP, GGT, and TBiL levels were similar to those of ALT (*P < 0.05 vs. LT group). **(C)** TLR4 levels in the liver tissues of each group. Among them, the level on POD 7 in the LT group was the highest. **(D)** The expression of *Tlr4* mRNA in the liver tissue of each group. The hepatic *Tlr4* mRNA level was the highest on POD 7 in the LT group and the lowest on POD 7 in the HBM group. **(E)** Changes in the levels inflammatory factors IL-1β, TNF-α, IL-6, and IL-10 in serum. On POD 1, the LT group had the highest levels of IL-1β, TNF-α, and IL-6. On POD 7, the levels of TNF-α, IL-1β, and IL-6 were still higher in the LT group, while their levels in the HBM group decreased the most. *P < 0.05. **(F)** Changes of *Il1b, Tnfa, Il6*, and *Il10* mRNA in serum. *P < 0.05, **P < 0.01, ***P < 0.001, ****P < 0.001.

Liver histology showed lipid droplets in more than 90% of hepatocytes in the Sham group, indicating mixed steatosis. On POD 1, hepatic sinusoids in the LT group were narrowed, with obvious congestion or even necrosis, and fewer lipid droplets and severe infiltration of inflammatory cells were seen. In the BM and HBM groups, obvious congestion of hepatic sinusoids, vesicular steatosis, and inflammatory cell infiltration were observed, but to a lesser extent than in the LT group. On POD 7, large areas of liver tissue necrosis were seen in the LT group; whereas, in the BM group, there was mild steatosis, and the liver cords were neatly arranged. In the HBM group, the liver cords were neatly arranged without lipid droplets or inflammatory cell infiltration (Figure 2A).

Liver function tests showed that on POD 1, ALT, AST, ALP, GGT, and TBiL levels increased rapidly in the LT group (Figure 2B); the BM and HBM groups had lower levels of these markers than the LT group, but higher than those in the Sham group (*P* < 0.05). On POD 7, the levels of AST and ALT in the LT group decreased, but ALP, GGT, and TBiL levels continued to increase; ALB was in a state of decline (*P* < 0.05). All indexes in the BM and HBM groups were improved compared with those in the LT group, and the improvement in the HBM group was significantly greater than that in the BM group on POD 7 (*P* < 0.05).

These results suggested that MSCs can improve the histology and functions of steatotic liver grafts, and the effect of HO-1/BMMSCs was better than that of BMMSCs alone over time.

For inflammation, the level of TLR4 in the liver tissue in the LT group was the highest after liver transplantation, and on POD 7 was higher than that of POD 1; the level of TLR4 in the HBM group was the lowest on POD 7 (Figure 2C). The qRT-PCR results also confirmed that the *Tlr4* mRNA level in the LT group was significantly increased (Figure 2D). Detection of inflammatory factors showed that on POD 1, compared with those in the Sham group, the levels of IL-1β, TNF-α, and IL-6 in the LT group were the highest; while the levels of IL-1β, TNF-α, and IL-6 in the BM and HBM groups increased, but not significantly compared with those in the LT group. The level of IL-10 increased; however, there was no statistical difference between the BM and HBM groups (*P* > 0.05) (Figure 2E). On POD 7, the levels of TNF-α, IL-1β, and IL-6 in the LT group were still high. Compared with those in the LT group, the levels of TNF-α, IL-1β, and IL-6 in the BM and HBM groups decreased, with the decrease being most significant in the HBM group (*P* < 0.05). The level of IL-10 increased most significantly in the HBM group (*P* < 0.05). The mRNA results showed the same patterns (Figure 2F). Hence, inflammatory factors are involved in the pathological process after liver transplantation, and MSCs reduced the hepatic inflammatory response after steatotic liver transplantation and prolonged postoperative time. The anti-inflammatory function of HO-1/BMMSCs was significantly better than that of BMMSCs.

### The Effect of HO-1/BMMSCs on the Structure and Function of Recipient Intestines

**Figure 3:**
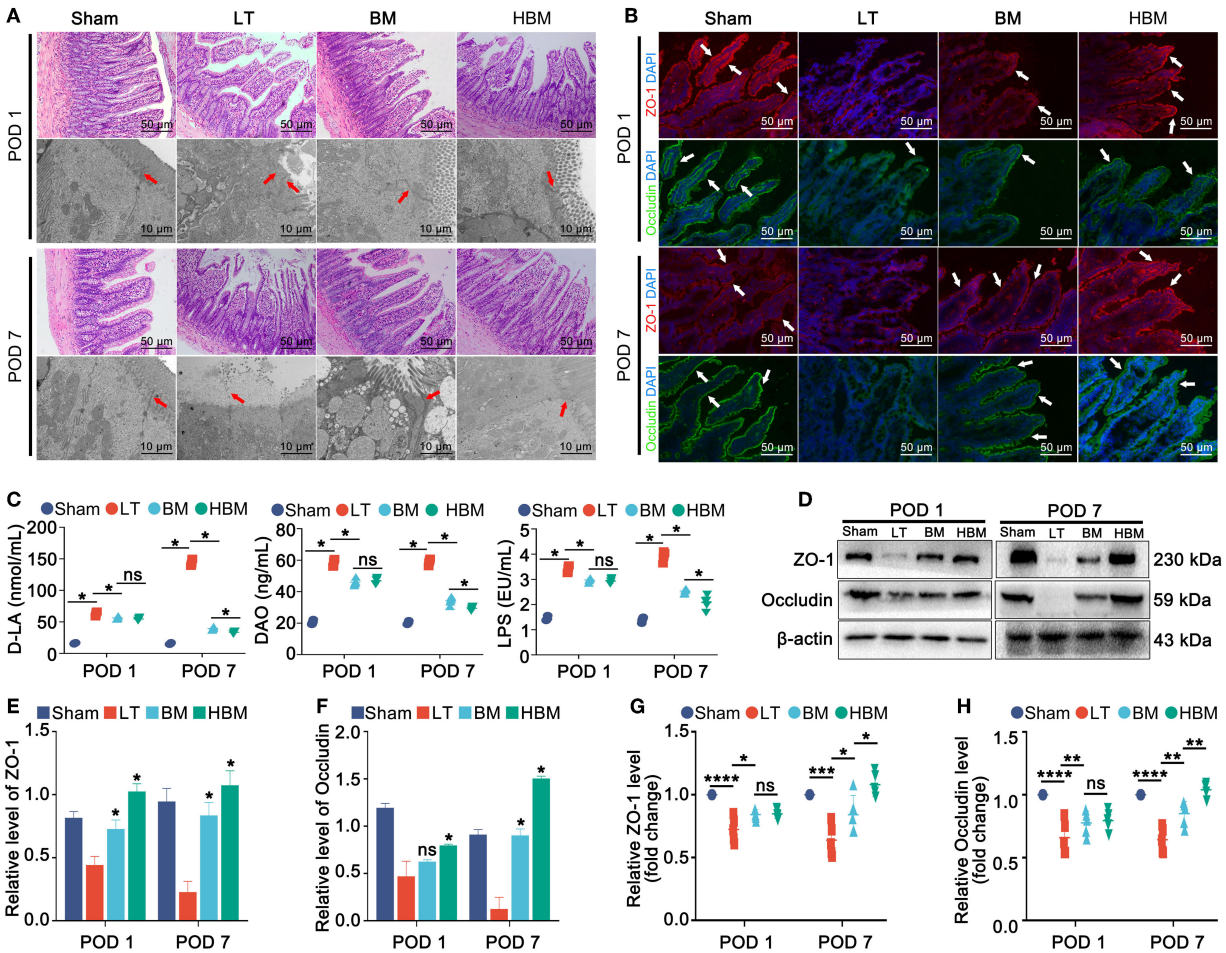
Changes in intestinal histology, ultrastructure, and permeability of the recipients. **(A)** Histopathology of steatotic donor liver recipient rats at different time points after surgery (×200). **(B)** Immunofluorescence of intestinal tight junction proteins ZO-1 and Occludin (indicated by a white arrow; ×200). **(C)** Changes in intestinal permeability. On POD 1, plasma D-LA (63.17 ± 3.74 nmol/mL), DAO (58.56 ± 2.42 ng/mL), and LPS (3.42 ± 0.15 EU/mL) in the LT group were significantly increased (*P* < 0.05); on POD 7, D-LA (144.10 ± 5.42 nmol/mL) and LPS (3.94 ± 0.20 EU/mL) in the LT group continued to increase; while D-LA, DAO, and LPS in the BM group showed a stable trend, but decreased in the HBM group (*P* < 0.05). **(D–H**) Protein and mRNA expression levels of ZO-1 and Occludin. *P < 0.05, **P < 0.01, ***P < 0.001, ****P < 0.001.

In terms of intestinal histology, the intestinal villi in the Sham group were neat. In the LT group, on POD 1, the intestinal villi were obviously destroyed, the villi were broken, and the villus epithelium was detached; on POD 7, the villous epithelium was still obviously detached. In the BM and HBM groups, severe detachment of the villous epithelium was seen on POD 1; on POD 7, the intestinal villi were regularly arranged, and there was no intestinal villus breakage or detachment of the villous epithelium (Figure 3A). In the Sham group, the microvilli of the intestinal epithelial cells were neat, the tight junctions between the intestinal epithelial cells were intact, desmosomes were present, and the intercellular space had not widened. However, in the LT group, on POD 1, the microvilli of the intestinal mucosal epithelial cells became short, sparse, and broken, and showed serious cell loss; on POD 7, intestinal epithelial cell necrosis was seen. In the BM and HBM groups, the intact tight junctions were still visible on POD 1, and on POD 7, the tight junctions were more intact.

On POD 1, the expression levels of the tight junction proteins ZO-1 and Occludin in the LT group were low and interrupted, while their expression levels in the BM and HBM groups were slightly higher than those in the LT group, and interruption was not obvious. On POD 7, the tight junction proteins ZO-1 and Occludin in the LT group were still at a low level, even lower than that of POD 1; whereas, their expression levels in BM and HBM groups were higher than those on POD 1, and the HBM group showed a more significant increase than the BM group (Figure 3B).

In terms of intestinal function, the plasma D-LA, DAO, and LPS levels in each group increased significantly on POD 1, with the largest increase being observed the LT group (*P* < 0.05), indicating that the LT group had increased intestinal permeability. There was no significant difference between the BM and HBM groups (*P* > 0.05). On POD 7, D-LA and LPS levels had increased further in the LT group, and DAO was in a stable state; while the D-LA, DAO, and LPS levels in BM group had stabilized, and those in the HBM group showed a downward trend (*P* < 0.05) (Figure 3C).

Hence, after liver transplantation with steatotic liver grafts, the intestinal mucosal structure was destroyed and the intestinal permeability continued to increase. MSCs could sightly improve intestinal permeability. Over time, the effect of HO-1/BMMSCs on intestinal permeability was better.

The detection of proteins and mRNAs showed that on POD 1, the expression of tight junction protein was the highest in the Sham group and the lowest in the LT group (*P* < 0.05). Among the groups, there was no difference in expression between the BM and HBM groups. On POD 7, their expression levels in the LT group remained the lowest among the groups; whereas, their expression levels in the BM and HBM groups increased, with the HBM group showing the most significant increase (*P* < 0.05; Figures 3D–F). The mRNA results showed the same patterns (Figures 3G,H; *P* < 0.05).

Hence, after liver transplantation with steatotic liver grafts, the intestinal mucosal structure is destroyed and the expression of tight junction proteins is reduced. Meanwhile, MSCs can reduce the damage to the recipients' intestinal tissue and promote the recovery of the recipients' intestinal tight junction proteins. The effect of HO-1/BMMSCs was better than that of BMMSCs over time.

### Effects of HO-1/BMMSCs on Injured Intestinal Epithelial Cells and Steatotic Hepatocytes *in vitro*

**Figure 4:**
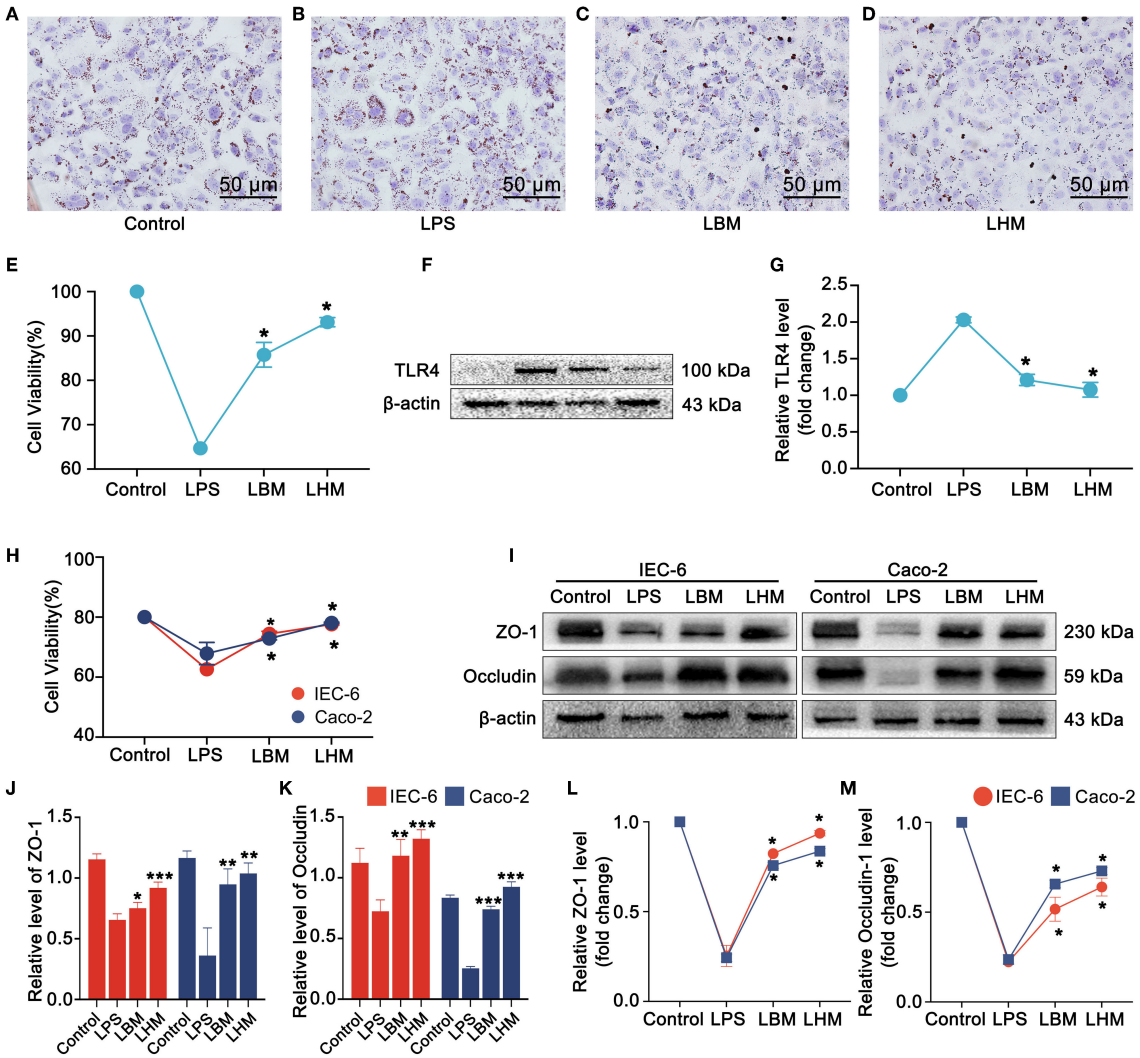
HO-1/BMMSCs repair LPS-injured steatotic hepatocytes and intestinal epithelial cells. **(A)** Normal IAR-20 cells with steatosis, stained with oil red (×200). **(B)** LPS-stimulated steatotic IAR-20 cells with increased positive oil red staining. **(C)** After LPS-stimulated steatotic IAR-20 cells were co-cultured with BMMSCs, the positive staining of oil red decreased significantly. **(D)** After LPS-stimulated steatotic IAR-20 cells were co-cultured with HO-1/BMMSCs, the positive oil red staining decreased significantly. **(E)** Cell viability of steatotic IAR-20 cells in each group. **(F)** Changes of TLR4 protein levels in steatotic IAR-20 cells in each group. **(G)** Expression levels of *Tlr4* mRNA in steatotic IAR-20 cells in each group. **(H)** Cell viability of IEC-6 and Caco-2 cells in each group. **(I–K)** Protein levels of tight junction proteins (ZO-1, Occludin) in each group. **(L,M)** mRNA levels of claudin (*Zo1, Ocln*) in each group. *P < 0.05 vs. LT group, **P < 0.01 vs. LT group, ***P < 0.001 vs. LT group, ****P < 0.0001 vs. LT group.

In the steatosis IAR20 cell model, after stimulation by LPS on steatotic cells (Figure 4A), the degree of steatosis increased (Figure 4B), while the degree of steatosis decreased significantly after co-culture with BMMSCs and HO-1/BMMSCs (Figures 4C,D). LPS stimulation decreased cell viability (Figure 4E) and increased TLR4 protein and mRNA levels (Figures 4F,G). After co-culture with BMMSCs and HO-1/BMMSCs, cell viability increased significantly, and the level of TLR4 was decreased. These results indicated that MSCs could reduce the degree of steatosis and inhibit the TLR4 inflammatory response induced by LPS.

After LPS acted on IEC-6 and Caco-2 cells for 24 h, the activity of intestinal epithelial cells decreased significantly (Figure 4H), and tight junction protein (ZO-1 and Occludin) levels were significantly reduced (Figures 4I–M). Under the indirect effect of BMMSCs and HO-1/BMMSCs, the viability of IEC-6 and Caco-2 cells increased significantly (*P* < 0.05), and the protein and mRNA expressions of tight junction proteins ZO-1 and Occludin also increased. This suggested that MSCs can repair damaged intestinal epithelial cells through paracrine action, improve cell viability, and enhance the expression of tight junction proteins.

### Effects of HO-1/BMMSCs on the Intestinal Microbiota of Recipients

To further explore whether the effect of HO-1/BMMSCs in repairing steatotic liver grafts is related to the intestinal microbiota, we investigated the changes in the gut microbiota of recipient rats under different conditions using 16S rRNA gene sequencing.

#### The Predominant Bacteria of the Recipients in all Groups

**Figure 5:**
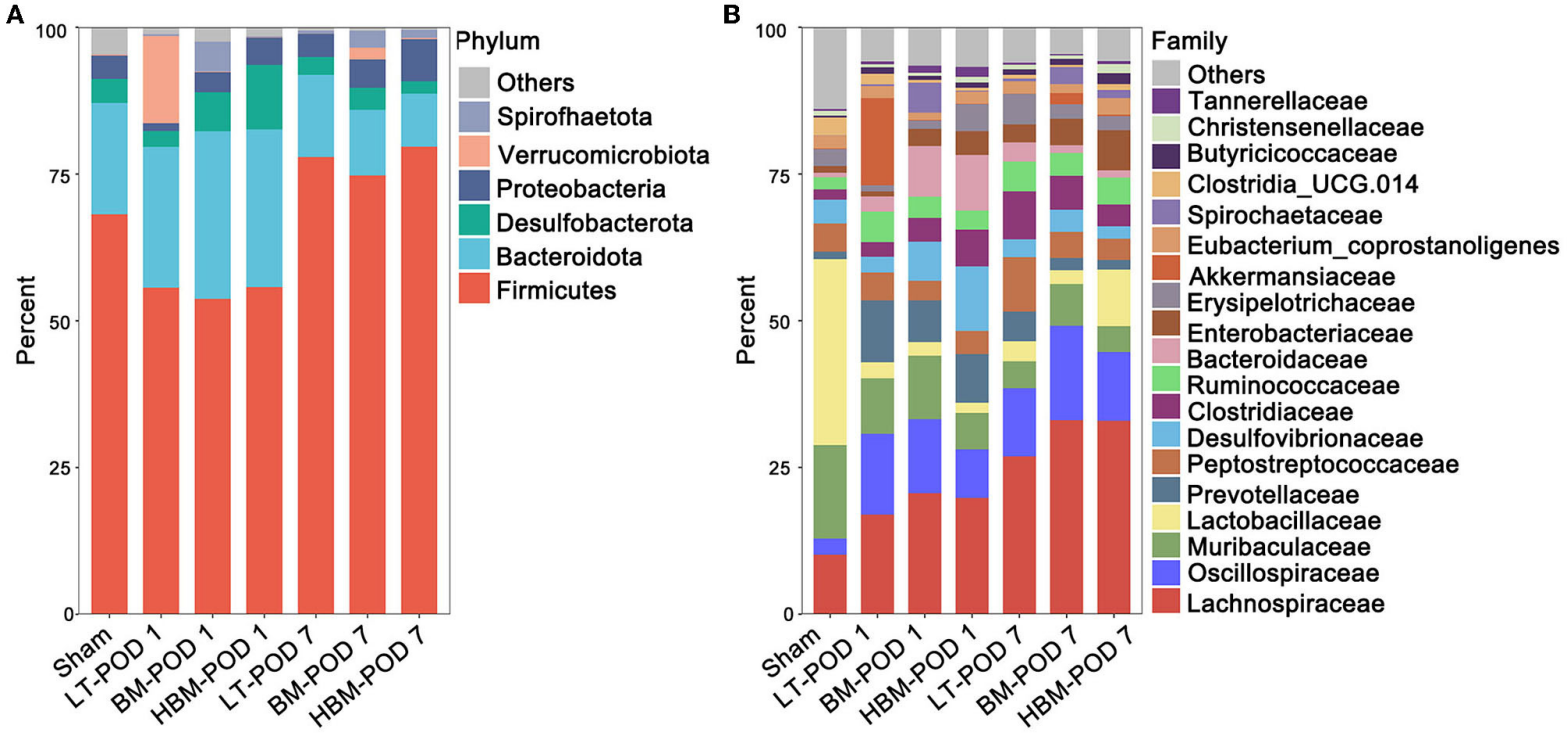
Changes in intestinal microbiota in the recipients. **(A)** Species composition and abundance of each group at the phylum level. *Firmicutes* phylum levels increased significantly on POD 7, and *Bacteroidota* phylum levels decreased significantly on POD 7. **(B)** Species composition and abundance of each group at the family level. Under the function of MSCs, the abundance of the *Lachnospiraceae* family increased significantly on POD 7.

At the phylum level, *Firmicutes, Bacteroidota, Desulfobacterota, Proteobacteria, Verrucomicrobiota*, and *Spirochaetta* had the highest abundance in all groups. *Firmicutes*/*Bacteroidota* decreased on POD 1, while *Firmicutes*/*Bacteroidota* increased on POD 7 (Figures 5A,B). At the family level, *Lachnospiraceae, Oscillospiraceae, Muribaculaceae, Lactobacillaceae*, and *Prevotellaceae* were the most abundant microbiota in all groups.

#### Species Diversity

Rarefaction and Specaccum curves (Supplementary Figures 2A,B) indicated that the amount of sample sequencing data was reasonable and could be analyzed. Alpha diversity reflects the species diversity within each group. Shannon's index of alpha diversity showed that the number of OTUs in the Sham group was significantly lower than that in the other groups, especially on POD 7 (*P* < 0.05) (Supplementary Figure 2C). This suggested that liver transplantation with steatotic live grafts led to an increase in the species diversity of the recipient intestinal microbiota. Beta diversity aims to compare how similar in different samples are in terms of species diversity. Based on the PCA analysis, compared with the Sham group, the other groups were farther away from it (Supplementary Figure 2D), and the similarity of the beta diversity of intestinal microbiota decreased significantly, indicating that liver transplantation with steatotic liver grafts significantly changed the intestinal microbiota composition.

#### Differences in the Microbiota in Each Group

**Figure 6:**
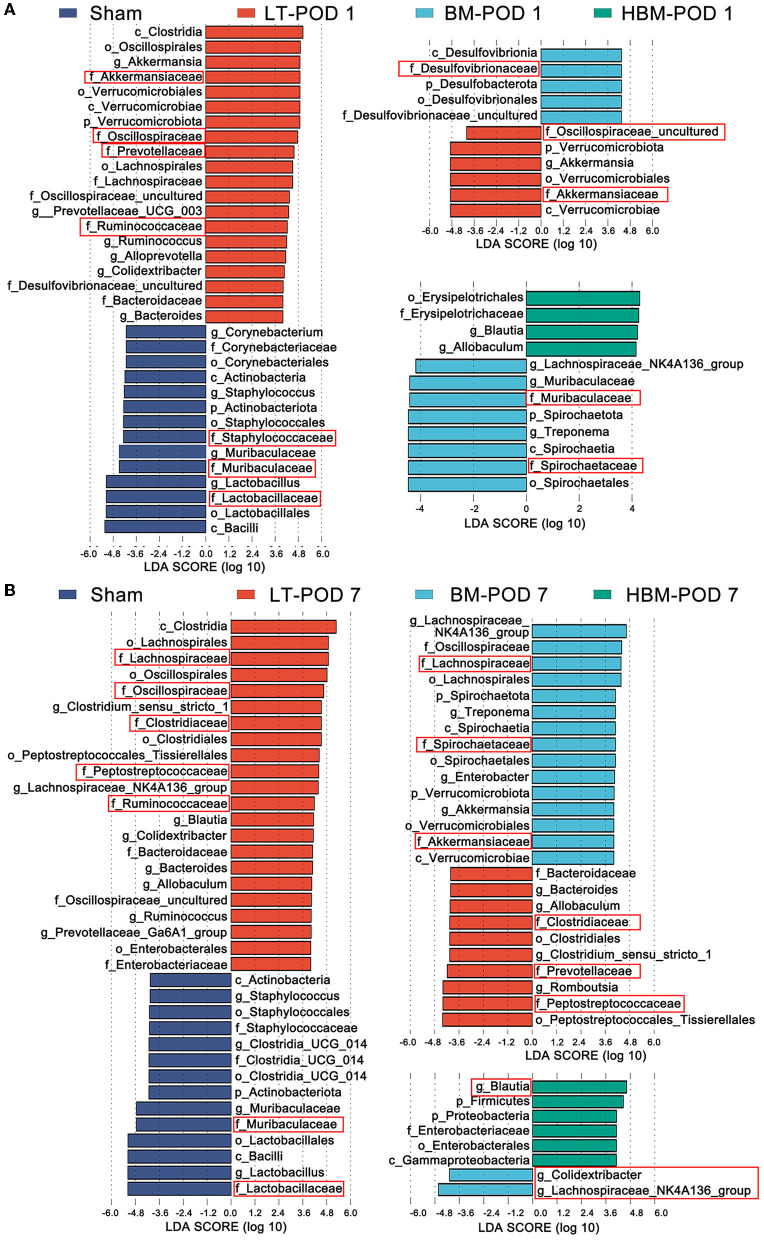
**(A)** Species abundances of different species between groups in the Lefse analysis on POD 1; the red box shows the microbiota with obvious differences at the family level. **(B)** Lefse analysis of the histogram of the abundance of different species among the groups on POD 7; the red box shows the flora with obvious differences at the family level.

We used Lefse (Line Discriminant Analysis (LDA) Effect Size) analysis (LDA Score greater than the set value of 4.0) to screen out species with significant differences in abundance between groups (biomarkers). At the family level, on POD 1, compared with the Sham group, the abundances of *Akkermansiaceae, Oscillospiraceae, Prevotellaceae*, and *Ruminococcaceae* in the LT group were higher; while the abundances of *Lactobacillaceae, Muribaculaceae*, and *Staphylococcaceae* decreased significantly (*P* < 0.05). Compared with those in the LT group, the BM group showed an increased abundance of *Desulfovibrionaceae*, while *Akkermansiaceae* and *Oscillospiraceae* had lower abundances (*P* < 0.05). Compared with those in the BM group, the HBM group had lower abundances of *Spirochaetaceae* and *Muribaculaceae* (Figure 6A). This suggested that on POD 1, the increased intestinal microbiota of BMMSCs and HO-1/BMMSCs is mainly *Desulfovibrionaceae*, which might be involved in the repair of intestinal mucosal function.

On POD 7, the abundances of *Lachnospiraceae, Oscillospiraceae, Clostridiaceae, Peptostreptococcaceae*, and *Ruminococcaceae* were higher in the LT group compared with those in the Sham group, while the abundances of *Lactobacillaceae* and *Muribaculaceae* remained low (*P* < 0.05). Compared with those in the LT group, the abundances of *Lachnospiraceae, Spirochaetaceae*, and *Akkermansiaceae* increased in the BM group, while the abundances of *Peptostreptococcaceae, Prevotellaceae*, and *Clostridiaceae* decreased; *Oscillospiraceae* (*Colidextribacter*) richness also decreased (*P* < 0.05; Figure 6B). These results suggested that on POD 7, MSCs mainly increased the levels of butyrate-producing bacteria, such as *Lactobacillaceae* and *Akkermansiaceae*, and bacteria that improved fat metabolism, and HO-1/BMMSCs further increased the levels butyrate-producing bacteria, such as *Blautia*.

### The Intestinal Microbiota Is Involved in the Protective Effect of HO-1/BMMSCs on Recipients

**Figure 7:**
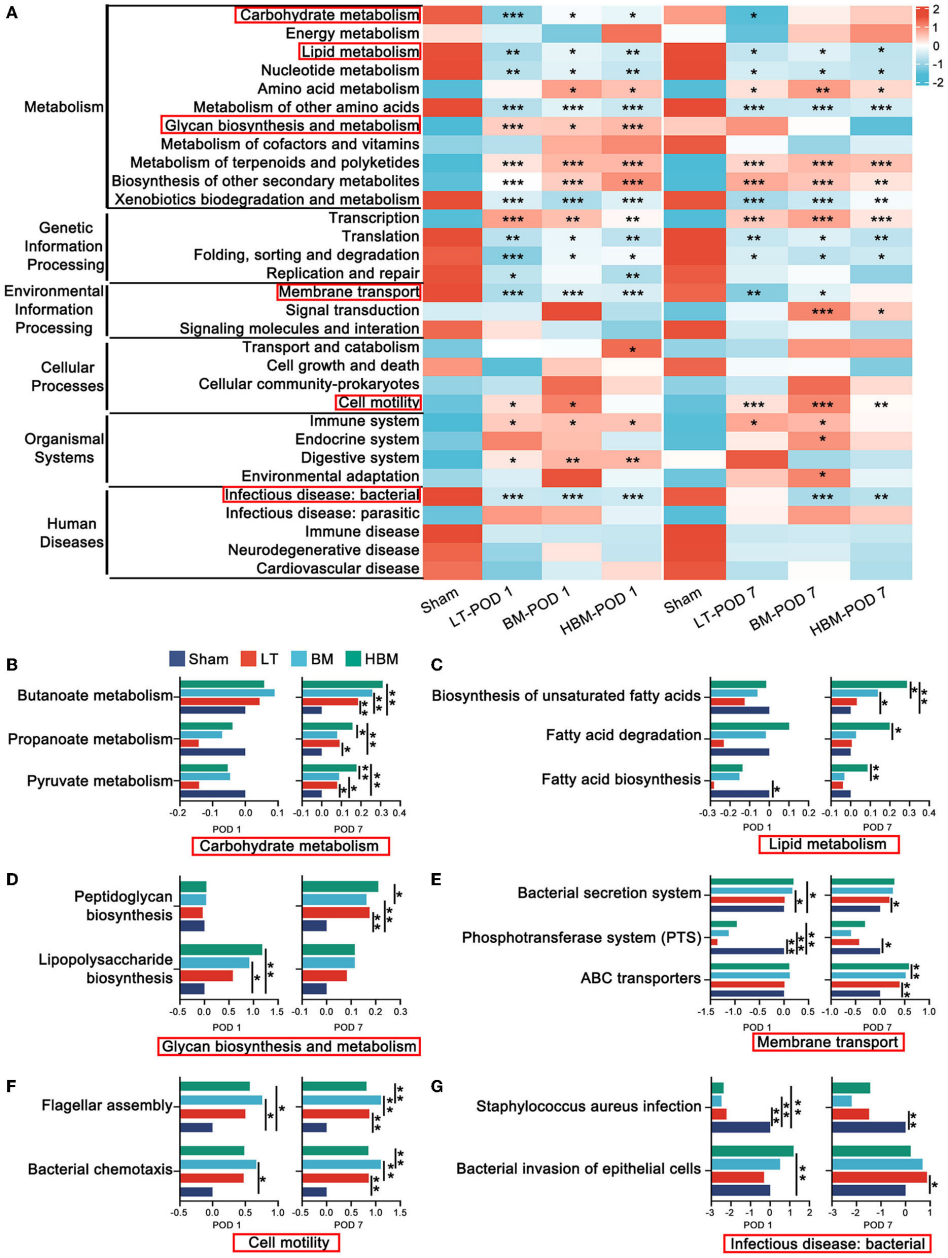
PICRUSt functional prediction of changes in the intestinal microbiota. **(A)** Six main pathways at level 1 and 31 main pathways at level 2. Among them, carbohydrate metabolism, lipid metabolism, glycan biosynthesis and metabolism, membrane transport, cell motility, and bacterial infection disease play an important role in the repair process involved in the steatotic transplanted liver (the important secondary pathways are in the red box). **(B)** The expression levels of genes related to Pyruvate metabolism, Propanoate metabolism, and Butanoate metabolism in the carbohydrate metabolism groups were highest in the HBM group. **(C)** The expression levels of genes related to Fatty acid degradation and the Biosynthesis of unsaturated fatty acids in each group in fat metabolism were the highest on POD 7 in the HBM group. **(D)** In glycan biosynthesis and metabolism, the expression levels of genes in the Lipopolysaccharide biosynthesis groups showed the most significant decrease in the HBM group. **(E)** Membrane transport pathways in each group were significantly enhanced in the BM and HBM groups. **(F)** For flagellar assembly in cell motility, the expression levels of genes related to Bacterial chemotaxis in each group were the strongest on POD 7 in the BM group. **(G)** The expression levels of genes related to Bacterial invasion of epithelial cells in each group of bacterial infection disease were the highest level on POD 7 in the LT group. *P < 0.05, **P < 0.01, ***P < 0.001, ****P < 0.001.

For the intestinal microbiota, the PICRUSt function prediction (Figure 7A) results showed the pathway changes of each group for carbohydrate metabolism, lipid metabolism, glycan biosynthesis and metabolism, membrane transport, cell motility, and infectious disease: bacterial. For short-chain fatty acid metabolism pathways: Pyruvate metabolism, Propanoate metabolism, and Butanoate metabolism were significantly enhanced on POD 7 in the HBM group, and were higher than those of the BM group (*P* < 0.05), whereas on POD 7, there were no significant differences between the BM and LT groups (Figure 7B). Fatty acid degradation in the Fatty acid metabolism pathway and the Biosynthesis of unsaturated fatty acids pathway were significantly enhanced on POD 7 in the HBM group (*P* < 0.05; Figure 7C), and were significantly higher than those of the BM group. However, there was no statistical difference between the BM and LT groups. For pathways associated with pathogen-related molecular patterns: Peptidoglycan biosynthesis was significantly enhanced on POD 7 in the LT, BM, and HBM groups (Figure 7D); Lipopolysaccharide biosynthesis was highest on POD 1 in HBM group, and significantly decreased on POD 7 in the BM and HBM groups, and declined the most in the HBM group (Figure 7D). In Membrane transport pathways related to bacterial pathogenicity, the Bacterial secretion system was significantly increased on POD 7 in the LT, BM, and HBM groups. For the pathways related to bacterial uptake of nutrients, Phosphotransferase system (PTS) and ABC transporters were significantly increased in BM and HBM groups on POD 7, and were significantly enhanced in the HBM group compared with that in the BM group (*P* < 0.05; Figure 7E). For pathways related to bacterial motility: Flagellar assembly and Bacterial chemotaxis were strongest on POD 7 in the BM group. Meanwhile, these two bacterial motility pathways were enhanced in the LT and HBM groups compared with those in the Sham group, but there was no statistical difference between them (Figure 7F). Among bacterial infectious diseases, *Staphylococcus aureus* infection was significantly decreased in the LT, BM, and HBM groups and Bacterial invasion of epithelial cells was highest on POD 7 in the LT group; however, there was no statistical difference between the BM and HBM groups (Figure 7G).

## Discussion

Although steatotic donor livers account for a large proportion of the donor liver pool, the risk of post-transplantation recipient mortality, early primary graft dysfunction, and primary nonfunction in recipients with steatotic liver grafts is significantly higher than that of non-steatotic livers grafts (Mccormack et al., 2011; Croome et al., 2020; Chen et al., 2021). Therefore, acquiring an understanding of the damage repair mechanism of steatotic liver grafts is significant to improve and optimize the prognosis of steatotic liver transplantation. In this study, we investigated whether the intestinal microbiota plays an important role in the repair of steatotic liver grafts by HO-1/BMMSCs and explored a new way to repair steatotic liver grafts.

MSCs play an important role in the repair and regeneration of tissue and organ damage and good results have been achieved in organ transplantation and repair (Yang et al., 2014; Yin et al., 2017, 2019; Cao et al., 2020; Tian et al., 2021). Our research group has verified that HO-1-modified BMMSCs have anti-inflammatory, anti-oxidative, anti-apoptotic, and delayed rejection functions in transplant recipients, and are more effective than BMMSCs alone. Activity in the diseased part is related to prolonging its duration of function (Cao et al., 2020; Tian et al., 2021). However, there is no relevant research on the effect of MSCs on steatotic liver transplantation. This study aimed to observe the effect of HO-1/BMMSCs on recipients after steatotic liver transplantation in rats, and on this basis, to observe the effect of the intestinal microbiota.

Hepatic sinusoidal microcirculation disturbance is one of the main reasons why steatotic liver grafts are more susceptible to ischemia-reperfusion injury, which causes primary graft nonfunction and other complications (Teramoto et al., 1993). Compared with a normal liver, the degree of stenosis of hepatic sinusoids in steatotic liver grafts can reach 50% (Ijaz et al., 2003). In this experiment, we established a stable steatotic liver transplantation model and directly infused MSCs into the portal vein. On POD 1, we found that the degree of liver congestion, steatosis, and inflammatory cell infiltration decreased in the MSC groups. On POD 7, the degree of repair exerted by HO-1/BMMSCs to the steatotic liver grafts was significantly better than that of BMMSCs, i.e., there was no congestion in the hepatic sinusoids, the fatty infiltration almost disappeared, and the degree of inflammatory cell infiltration was significantly reduced. Meanwhile, HO-1/BMMSCs could significantly inhibit the expression of TLR4 and pro-inflammatory factors in steatotic livers grafts. This demonstrated that HO-1/BMMSCs have more advantages in improving the prognosis and survival rate of steatotic liver transplant recipients in rats compared with BMMSCs alone.

D-LA is a metabolite of various intestinal bacteria and cannot be rapidly degraded. DAO is mainly concentrated in the small intestinal mucosal villi. LPS is a major component of gram-negative bacteria and is released after enterobacterial cell death. Therefore, plasma D-LA, DAO, and LPS levels might partly reflect changes in gastrointestinal permeability and intestinal mucosal barrier function (Cai et al., 2019). During steatotic liver transplantation, portal vein blood flow is blocked (30 min), and severe congestion of the mesentery, ischemia, and hypoxia of the intestinal wall tissue, and further damage to the intestinal mucosal structure, occur, i.e., villi breakage and detachment of the villus epithelium (Deng et al., 2021a,b). We found that plasma D-LA, DAO, and LPS levels increased significantly after liver transplantation with steatotic liver grafts compared with those without surgery, while the levels of ZO-1 and Occludin, the key proteins of intestinal tight junctions, decreased significantly, indicating that there might be dysbacteriosis and intestinal mucosal damage. When MSCs were administered, the levels of D-LA, DAO, and LPS decreased significantly, while ZO-1 and Occludin levels increased significantly, and the effect of HO-1/BMMSCs was the most obvious with time. This indicated that HO-1/BMMSCs could reduce intestinal mucosal damage and protect intestinal permeability indirectly after the restoration of the sinusoidal microcirculation of the steatotic liver grafts, and their effects were more durable than those of BMMSCs.

*In vitro* experiments showed that LPS could reduce the activity of steatotic IAR20 cells, aggravate the degree of steatosis, and increase the expression of TLR4. Moreover, LPS reduced the expression of tight junction proteins ZO-1 and Occludin, and increased intestinal permeability. However, after treatment with MSCs, the activity of IAR20 cells increased, the degree of cellular steatosis was decreased, and the expression of TLR4 decreased significantly. The activities of IEC-6 and Caco-2 cells increased, and the expression of tight junction proteins also increased. These results indicated that in steatotic liver cells, MSCs could reduce the degree of steatosis and the expression of TLR4, which would decrease the inflammatory response of the transplanted liver. Moreover, MSCs also reduced the damage caused by LPS on intestinal epithelial cells through a paracrine effect.

The intestinal microbiota plays an important role in the alleviation of steatotic liver grafts (Bäckhed et al., 2004; Le Roy et al., 2013; Safari and Gérard, 2019). Modulating the gut microbiome is a promising therapeutic approach in nonalcoholic fatty liver disease (NAFLD; Lee et al., 2020). Probiotics has been used to prevent and treat NAFLD (Khan et al., 2021). The interplay between the gut microbiota, the intestinal barrier, the immune system, and the liver is complex (Martín-Mateos and Albillos, 2021). To further explore whether the recipient's intestinal microbiota has any effect on the recipients after steatotic liver transplantation, we investigated the intestinal microbiota of the recipients on POD 1 and POD 7. The results showed that the pathogenic bacteria in the recipients' intestinal microbiota increased significantly in the LT group over time, while in the BMMSCs and HO-1/BMMSCs groups, there were significant increases in the number of bacteria with increased lipid metabolism and butyrate production.

On POD 1, the abundance of anaerobic bacteria *Akkermansiaceae* increased significantly after steatotic liver transplantation. *Akkermansiaceae* use mucins in the mucus layer of the intestine as a source of carbon and nitrogen. *Akkermansiaceae* are closely related to the intestinal health of the body and pathological states such as obesity (Plovier et al., 2017), and are involved in the repair of damaged intestinal epithelium, antagonize pathogenic bacteria, and consolidate the function of new intestinal epithelium (Reunanen et al., 2015). Rao et al. (2021) found that obese mice treated with *A. muciniphila* eliminated hepatic steatosis, inflammation, and liver injury through regulating the metabolism of L-aspartate. However, on POD 1 after steatotic liver transplantation, the intestinal villi were significantly disrupted, edema and the infiltration of inflammatory cells led to a significant increase in *Akkermansiaceae* under conditions of low nutritional status. Intestinal epithelial cells were damaged, tight junctions were destroyed, intestinal resistance was weakened, bacterial metabolism was weakened, and overgrowth occurred, such that the increased abundance of *Akkermansiaceae* did not play a significant protective role in the intestinal mucus layer, but instead promoted the decomposition of the mucus layer. Inflammatory bacteria (e.g., *Ruminococcus, Bacteroides*, and *Erysipelotrichaceae*; Kaakoush, 2015; Hall et al., 2017; Miyauchi et al., 2020; Henke et al., 2021) enhance the invasive intestinal epithelial cell response, which in turn allows LPS and a large number of bacterial metabolites to enter the portal vein. This indicated that the increase of bacterial diversity after steatotic liver transplantation did not protect the recipients, but because of the enhanced ability of bacteria to invade the epithelium, the metabolites entered the liver through the portal vein and aggravated the inflammatory response of the steatotic liver grafts.

Under the function of MSCs, the levels of butyrate-producing bacteria, such as *Desulfovibrionaceae, Lachnospiraceae*, and *Blautia*, increased; the levels of bacteria that improved lipid metabolism, such as *Akkermansiaceae*, increased; and the levels of obesity-causing bacteria, such as *Peptostreptococcaceae, Prevotellaceae*, and *Erysipelotrichaceae*, decreased. Over time, the effect of HO-1/BMMSCs on the levels of these bacteria was the most significant. *Desulfovibrionaceae* produces LPS and is strongly associated with obesity and metabolic syndrome (Zhang et al., 2021); however, studies also have shown that the H_2_S produced by *Desulfovibrionaceae* is an important mediator of gastrointestinal mucosal defense repair and reduction of systemic inflammation (Wallace et al., 2018). Therefore, the abundance of *Desulfovibrionaceae* increased on POD 1, which might have a repairing effect on the intestinal mucosa, while on POD 7, its abundance decreased, resulting in less LPS, which can enter the liver and activate the TLR4 pathway (Xiong et al., 2017). In addition, the abundance of *Akkermansiaceae* increased and microbiota associated with obesity and hyperlipidemia (*Peptostreptococcaceae, Prevotellaceae*, and *Erysipelotrichales*) decreased (Castonguay-Paradis et al., 2020; Sookoian et al., 2020). These microbiota alterations could improve glucose tolerance, insulin resistance, and protect the intestinal mucosa (Plovier et al., 2017).

The phylum *Firmicutes* can metabolize dietary fiber to butyrate, and *Spirochaetes* and *Proteobacteria* are also potential butyrate-producing bacteria (Fu et al., 2019). Butyrate is not only an energy source for colon cells, but also regulates genes epigenetically by inhibiting histone deacetylases, modulates intestinal immunity and inflammatory responses (Singhal et al., 2021), maintains and improves intestinal function, plays a role in reducing obesity, and has an important role in improving insulin resistance (Koh et al., 2016; Morrison and Preston, 2016; Fu et al., 2019). More importantly, butyrate reduction was associated with reduced numbers of tight junctions and increased intestinal permeability (Tripathi et al., 2018). MSCs could significantly increase the abundance of *Lachnospiraceae* in the class *Clostridia*. *Lachnospiraceae* are important butyrate-producing bacteria (Berger et al., 2021), which not only produce butyrate, with multifaceted effects on host metabolism and immune function, but also generate peptide antibiotics and convert primary bile acids to secondary bile acids, which promote the microbial community's response to drug-resistant pathogens (Sorbara et al., 2020). HO-1/BMMSCs could also increase the abundance of the butyrate-producing bacteria, *Blautia* (Wang et al., 2020), over time. Therefore, under the action of MSCs, LPS production was reduced, bacterial metabolism was enhanced, especially the metabolism of lipid and butyrate production. The effect of HO-1/BMMSCs on these beneficial parameters was more obvious with time. In addition, the protective effect of the microbiota on intestinal epithelial tight junction proteins would reduce the chance of bacteria and their metabolites entering the portal vein, thereby reducing the inflammatory response of the steatotic liver grafts.

All of these results indicated that the liver microcirculation is impaired after steatotic liver transplantation. Although the portal vein is opened after transplantation, the portal vein pressure might still be high in the short term after transplantation; therefore, mesenteric congestion cannot be effectively relieved. However, MSCs can reduce the degree of steatosis, alleviate the inflammatory response of the liver, and restore the microcirculation of the donor liver as soon as possible, which might reduce the portal venous pressure, reduce mesenteric congestion, and maintain the integrity of the intestinal barrier function, which could regulate the intestinal microbiota, and reduce the abundance of pathogenic bacteria. Importantly, the effects of BMMSCs optimized with HO-1 were more pronounced and longer-lasting over time than those of BMMSCs alone. Thus, HO-1/BMMSCs can better improve lipid metabolism, increase butyrate-producing bacteria, and help to repair steatotic liver grafts.

## Conclusion

HO-1/BMMSCs can accelerate lipid metabolism, reduce the inflammatory response, and improve the degree of congestion and steatosis of the steatotic liver grafts. In addition, HO-1/BMMSCs can protect the intestinal epithelial barrier and regulate intestinal microbiota homeostasis, reduce LPS in the blood, and increase the abundance of bacteria that improve lipid metabolism and butyrate production, thereby exerting a protective effect on steatotic liver grafts. Thus, HO-1/BMMSCs have a beneficial effect on the long-term survival of recipients.

This study had limitations: there was no research on the production and excretion of bile acids, which are important because the metabolism of bile salts by the intestinal microbiota can regulate the consumption of intestinal fat and can also affect the composition of the intestinal microbiota. In addition, the specific changes of portal pressure and the role of liver Kupffer cells also require further study.

## Data Availability Statement

The data presented in the study are deposited in the NCBI SRA database, accession number: PRJNA817803.

## Ethics Statement

All animal experiments followed the Guidelines for the Care and Use of Laboratory Animals and were approved by the Animal Ethics Committee of Nankai University (Permit Number: 2021-SYDWLL-000331).

## Author Contributions

All authors have contributed to the study conception and design, commented on previous versions of the manuscript, and read and approved the final manuscript. HS conceived and designed experiments. MY, LL, HC, and HS conducted the experiments and obtained the results. WZ, LW, HZ, XT, and HS sorted and analyzed the results. MY and HS wrote the draft, extensively revised, formatted, and submitted versions of the manuscript.

## Funding

The work was supported by the National Natural Science Foundation of China (Nos. 82070639, 81670574, 81441022, and 81270528).

## Conflict of Interest

The authors declare that the research was conducted in the absence of any commercial or financial relationships that could be construed as a potential conflict of interest.

## Publisher's Note

All claims expressed in this article are solely those of the authors and do not necessarily represent those of their affiliated organizations, or those of the publisher, the editors and the reviewers. Any product that may be evaluated in this article, or claim that may be made by its manufacturer, is not guaranteed or endorsed by the publisher.
