# Supplementary material for: Intestinal Microbiota Participates in the Protective Effect of HO-1/BMMSCs on Liver Transplantation With Steatotic Liver Grafts in Rats
Source: Front Microbiol. 2022 Jun 10;13:905567. doi: 10.3389/fmicb.2022.905567 (PMC9226684; doi:10.3389/fmicb.2022.905567)
Supplement: Supplementary file 1 [file Data_Sheet_1.PDF]

Supplementary Material

Supplementary Figures

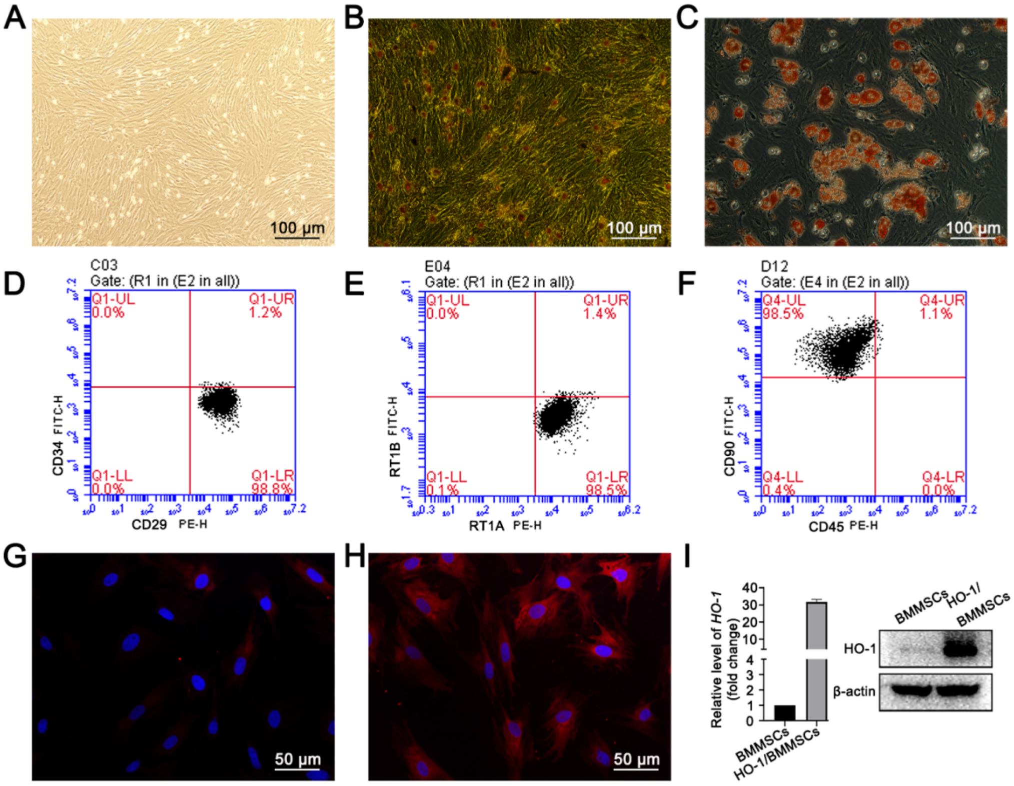

Figure S1: Characterization of HO-1/BMMSCs *in vitro* and detection of HO-1 expression. **A**. HO-1/BMMSCs are adherent and display a long spindle-shaped morphology. **B**. HO-1/BMMSCs show osteogenic differentiation *in vitro*, with von Kossa staining showing black calcium deposits. **C**. Adipogenic differentiation of HO-1/BMMSCs, oil red staining shows adipocytes. **D-F**. Surface Biomarker Identification. The results show that 98.8%, 98.5%, and 98.5% of the cells were positive for CD29, CD90, and RT1A, respectively, and 98.8%, 98.5%, and 98.5% were negative for CD34, CD45, and RT1B, respectively. The molecular biological properties of HO-1/BMMSCs had not changed. **G**. The expression of HO-1 in BMMSCs was identified by red fluorescence with weaker intensity. **H**. HO-1 expression in HO-1/BMMSCs (red fluorescence). The intensity of HO-1/BMMSCs was significantly higher than that of BMMSCs. **I**. The results of qRT-PCR and western blotting confirmed the expression of HO-1, and the expression level of HO-1 in HO-1/BMMSCs was significantly higher than that in BMMSCs.

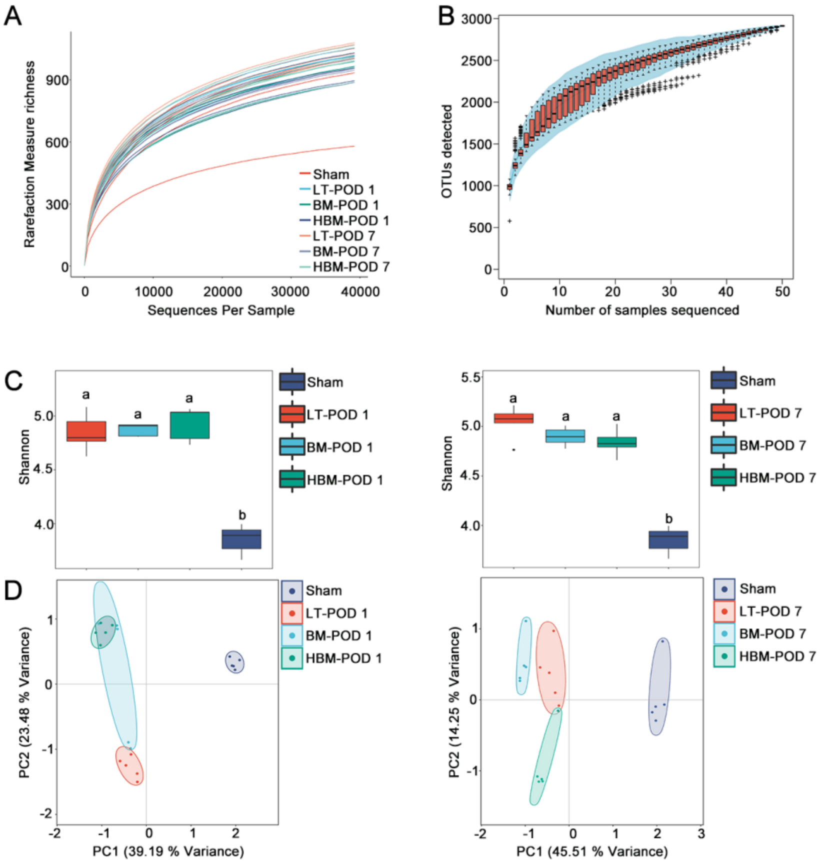

Figure S2: Species diversity. **A.** Rarefaction curve. The curve tends to be flat, indicating that the amount of sequencing data is reasonable. **B**. Specaccum curve. The curve tends to be flat, indicating that the species in this specimen did not increase significantly with the increase in the sample size, thus the sampling is sufficient for data analysis. **C**. Alpha diversity, reflecting species diversity within each group. The Shannon index shows that compared with the Sham group, the number of OTUs in the LT group increased significantly, and the species richness of the flora was higher. Different letters indicate statistical difference ($P < 0.05$). **D**. Beta diversity, which reflects the degree of similarity in species diversity among different samples. Compared with the Sham group, each group was farther away from the Sham group after transplantation, indicating that the species diversity changed significantly after transplantation.
